# Endostatin gene variation and protein levels in breast cancer susceptibility and severity

**DOI:** 10.1186/1471-2407-7-107

**Published:** 2007-06-22

**Authors:** Sabapathy P Balasubramanian, Simon S Cross, Jenny Globe, Angela Cox, Nicola J Brown, Malcolm W Reed

**Affiliations:** 1 Academic Surgical Oncology Unit, University of Sheffield, Sheffield, UK; 2 Academic Unit of Pathology, University of Sheffield, Sheffield, UK; 3 Institute of Cancer Studies, University of Sheffield, Sheffield, UK

## Abstract

**Background:**

Endostatin is a potent endogenous anti-angiogenic agent which inhibits tumour growth. A non-synonymous coding polymorphism in the Endostatin gene is thought to affect Endostatin activity. We aimed to determine the role of this Endostatin polymorphism in breast cancer pathogenesis and any influence on serum Endostatin levels in healthy volunteers. Endostatin protein expression on a breast cancer micro array was also studied to determine any relationship to genotype and to breast cancer prognosis.

**Methods:**

The 4349G > A (coding non-synonymous) polymorphism in exon 42 of the Endostatin gene was genotyped in approximately 846 breast cancer cases and 707 appropriate controls. In a separate healthy cohort of 57 individuals, in addition to genotyping, serum Endostatin levels were measured using enzyme linked immunosorbant assay (ELISA). A semi-quantitative assessment of Endostatin protein expression on immunostained tissue micro arrays (TMA) constructed from breast cancer samples of patients with genotype data was performed.

**Results:**

The rare allele (A) was significantly associated with invasive breast cancers compared to non-invasive tumours (p = 0.03), but there was no association with tumour grade, nodal status, vascular invasion or overall survival. There was no association with breast cancer susceptibility. Serum Endostatin levels and Endostatin protein expression on the tissue micro array were not associated with genotype.

**Conclusion:**

The Endostatin 4349A allele is associated with invasive breast cancer. The Endostatin 4349G > A polymorphism however does not appear to be associated with breast cancer susceptibility or severity in invasive disease. By studying circulating levels and tumour Endostatin protein expression, we have shown that any influence of this polymorphism is unlikely to be through an effect on the levels of protein produced.

## Background

Endostatin, a fragment of collagen 18-1α, was first identified in the conditioned medium of a hemangioendothelioma cell line as a potent inhibitor of angiogenesis and tumour growth [1]. The parent molecule releases Endostatin after proteolytic digestion by elastase and cathepsin L [2]. Endostatin inhibits endothelial cell proliferation and migration and induces apoptosis [3-5]. Several mechanisms have been postulated to explain the anti-angiogenic effects of Endostatin. One such mechanism is the high affinity of Endostatin for heparin explained by the presence of an extensive basic patch formed by 11 arginine residues. The interaction with heparin can interfere with the binding of basic fibroblast growth factor (bFGF) to cell surface heparan sulphate proteoglycans thereby inhibiting FGF growth signalling [6]. Alternatively, Endostatin can inhibit VEGF-induced migration of endothelial cells independent of the heparin binding epitope [7]. VEGF expression can also be reduced by anti-angiogenic factors such as Endostatin and Angiostatin resulting in impaired angiogenesis and reduced tumour growth [8]. In exponentially growing endothelial cells, many genes are down regulated by Endostatin including early response genes, cell cycle-related genes, and genes regulating apoptosis inhibitors, mitogen-activated protein kinases, focal adhesion kinase, G-protein-coupled receptors mediating endothelial growth, a mitogenic factor, adhesion molecules, and cell structure components [9]. The downregulation of pro-angiogenic genes and the upregulation of anti-angiogenic genes, together with the regulation of other genes unrelated to angiogenesis demonstrates that endostatin is involved in the complex interplay and extensive networking of the various signalling pathways in the microvascular endothelium [10].

The anti-tumour effect of Endostatin has been shown in a number of different solid tumours including melanoma, fibrosarcoma, renal cell carcinoma, mammary carcinoma and ovarian carcinoma [3]. In addition, in mice bearing Lewis lung carcinoma, T241 fibrosarcoma and B16F10 melanoma resistance to Endostatin suppression does not develop despite tumours being allowed to re-grow in between repeated treatment cycles [11].

Endostatin is one of the most potent and specific endogenous angiogenic inhibitors to enter clinical trials [12]. Several phase I studies have shown that the drug is well tolerated and without side effects [13-15].

The COL18A1 gene (which includes the Endostatin gene) was mapped in 1994 to the chromosomal region 21q22.3 by fluorescence *in situ *hybridization [16], with around 20 gene polymorphic variants identified[17]. One single nucleotide variant (G to A change) resulting in an aspartic acid to asparagine change (D104N) in exon 42 is in the encoding region for Endostatin and is a conserved site in both humans and mice. It is thought that the mutant molecule is stable but might impair Endostatin-induced angiogenesis inhibition by mechanisms that are still not clear [17]. Presence of the mutant allele results in a 2.5 times increased risk of prostate cancer [17], whereas in multiple myeloma there is no association with increased risk[18].

The aim of the study was to evaluate the Endostatin polymorphism in breast cancer susceptibility, severity, any association with serum Endostatin levels in healthy people and Endostatin protein expression on a breast cancer tissue micro array. The relationships between Endostatin protein expression and clinico-pathologic parameters in breast cancer were also evaluated.

## Methods

### Case and control selection

The design and methodology of the case control model has been previously described [19,20]. The cases include women diagnosed with breast cancer being followed up at the Royal Hallamshire Hospital in Sheffield and Rotherham District General Hospital and controls were recruited from asymptomatic women attending the Sheffield Breast Screening Service for regular screening mammograms. The study was restricted to white Caucasians, as there were insufficient individuals from other ethnic groups for meaningful analysis. The South Sheffield Research Ethics Committee approved the study [Ref. no. SS98/137] and informed written consent was obtained from all subjects. Demographic, environmental risk factors and family history data were recorded for all breast cancer cases and mammography screening controls, using a standard questionnaire. Pathological data (including tumour grade, lymph node status and presence of vascular invasion) were obtained from medical records and validated by an experienced histopathologist (SSC). Data on disease recurrence and overall survival were obtained from the hospital records and the Trent Cancer Registry. The data was entered by trained personnel and stored in a Microsoft Access database and maintained by a dedicated database administrator. The data was validated for all the records (by SPB and database manager).

### DNA extraction and Genotyping

**Figure 1:**
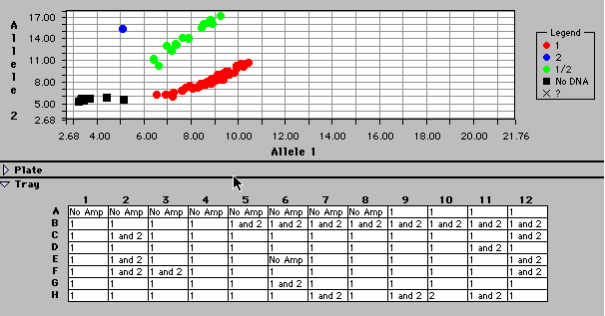
Endostatin genotyping results obtained from the Taqman Sequence Detection System. Samples are categorised into common homozygous or 1:1 (red), rare homozygous or 2:2 (blue) and heterozygous or 1:2 (green) groups.

From all participants, venous blood was collected in EDTA-vaccutainers and frozen at -20°C and later used for DNA extraction as described previously [21]. The sequence containing the polymorphism of interest [G > A nucleotide change resulting in D > N change in the amino acid sequence] was downloaded from GenBank (Accession numbers AL163302 and AF018081). The genomic sequence and coding sequence were aligned with each other using BLAST in NCBI website. The boundaries of the exons were noted and exon 42 together with the polymorphic site of interest was identified. An assay was designed to genotype the Endostatin polymorphism by the 5'nuclease PCR method, using the ABI/PE Biosystems Taqman™ system. Specifically designed primer and probe sequences included the forward primer (5'-GGCTCTGTTCTCAGGCTCTGA-3'), reverse primer (5'-GGCTCTCAGAGCTGCTCACA-3'), 6-carboxy-fluorescein (FAM)-labelled probe (5'-FAM-TCTCCTTT**A**ACGGCAAGGACGTCC-TAMRA-3') and 6-carboxy-4,7,2',7'-tetrechlorofluorescein (TET)-labelled probe (5'-TET-CTTCTCCTTT**G**ACGGCAAGGACG-TAMRA-3'). PCR amplification was carried out at a final volume of 25 μl. The final concentrations of the PCR constituents were 0.8 ng/μl genomic DNA template, 50 nM forward primer, 300 nM reverse primer, 50 nM FAM-labelled probe, 50 nM TET-labelled probe and 1 × (12.5 μl) Universal PCR mastermix (PE Biosystems) containing optimised buffer components and Rox reference dye. The PCR amplification cycle was 50°C for 2 min and 95°C for 10 min, followed by 40 cycles of 95°C for 15 seconds and 62°C for 1 min. Levels of FAM and TET fluorescence were determined and allelic discrimination was carried out using the ABI 7200 Sequence Detector (PE Biosystems). Quality control for the genotyping results was achieved by using only 72 of the 96 wells in each of the plates for the individual DNA samples subjected to PCR. Six to eight wells were allocated to 'no sample' controls, 'common homozygous' controls and 'rare homozygous' controls, in addition to re-evaluation of samples with indeterminate results. The common and rare homozygous controls included samples tested previously and demonstrated to be 'common homozygous' and 'rare homozygous' respectively. An example of the genotyping results obtained is shown in Figure 1.

### Serum Endostatin protein Estimation

Serum samples (in addition to genotyping data) were collected from a group of non-smoking healthy postmenopausal women (with no history of cancers) attending breast screening. Serum was collected in a serum separator tube, allowed to clot for at least 30 minutes and then centrifuged for 10 minutes. Serum samples were aliquoted into 4–5 eppendorf tubes and frozen at -20°C. Endostatin protein levels were determined using the Quantikine^® ^human Endostatin immunoassay kit (R&D Systems, Europe; catalog number DNST0). This assay employs the quantitative sandwich enzyme immunoassay technique using a mouse monoclonal antibody specific for human Endostatin.

### Endostatin Tissue Micro array

**Figure 2:**
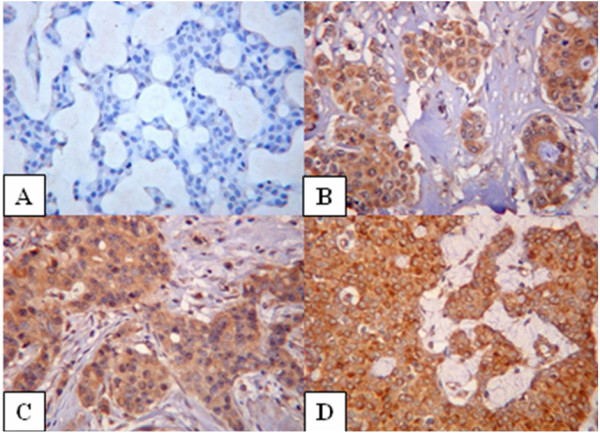
Negative (a), weak (b), moderate (c) and strong (d) Endostatin staining in invasive breast cancer. Magnification ×200.

A tissue micro array was constructed from archived paraffin embedded tumour samples of patients from the cancer cohort used in this study. From suitable areas on the tumour blocks, triplicate tissue cylinders with a diameter of 0.6 mm were punched using custom made precision instruments (Beecher Instruments Inc., Sun Prairie, US) and transferred into recipient paraffin blocks in a specific orientation. Five μm sections from the array blocks were dried, deparaffinized and rehydrated before blocking any endogenous peroxidase with a solution of 3% hydrogen peroxide in methanol. The sectioned tissue was then subjected to antigen retrieval by microwave treatment in 0.01 M sodium citrate solution.pH 6. This was followed by a standard immuno-histochemical staining procedure for Endostatin protein using goat anti-human Endostatin antibody (R&D systems Inc., UK) at a dilution of 1:4. This antibody was produced in goats immunized with purified, E. coli-derived, recombinant human Endostatin. Human tonsillar tissue was used as a positive control. The slides containing samples in triplicate were assessed for Endostatin staining (by an independent observer SSC who was blinded to the genetic and clinical data) and scored semi-quantitatively. Each core on the micro array was given a score from zero (no stain) to three (intense staining) depending on the intensity of the Endostatin staining by the tumour cells (Figure 2). The sum of all the three scores for each specimen was calculated to give a score on a scale of 0 to 9. For those with only two scores, the third was taken as the average of the first two and sum of the three was calculated. Specimens with one or no assessable cores were excluded.

### Data and statistical analyses

All data were entered initially into a Microsoft Access database and exported to SPSS (version 12.0.1 for Windows) for statistical analyses. Univariate non-parametric methods were used to test for associations between genotype and phenotype. Kaplan Meier curves and the log rank test were used for the survival analyses. Multivariate analyses and correction for multiple testing were not performed in view of the exploratory nature of this study. Results were considered statistically significant at p < 0.05.

## Results

### Endostatin polymorphism and breast cancer susceptibility and severity

The demographic characteristics and comparability of case and control cohorts have been reported previously [19,20]. Briefly, the case and control groups were all Caucasian and female. Table 1 shows the baseline characteristics of the population. There were no significant differences in the percentage of postmenopausal women, age at menarche and age at menopause between the cancer and control groups. The women in the control groups were however younger, were younger when first pregnant, had more children, were less likely to have a family history of breast cancer and were more likely to have smoked. Survival analyses were done in the cohort of women with invasive breast cancer who received surgery as a first line treatment and in whom follow up data was available. The median (interquartile range) follow up period for this group (n = 571) was 69 (48 and 102) months.

**Table 1:** Demographic characteristics of the 'breast cancer' and 'screening control' groups.

**CHARACTERISTICS**		**CASES**	**CONTROLS**	**P VALUE**
**Age**	N	847	712	P < 0.01^#^
	Median (IQR)	62 (54–70)	57 (53–61)	
**Ever Smoked**	N	844	712	P < 0.01^~^
	Yes	295 (35%)	316 (44.4%)	
	No	549 (65%)	396 (55.6%)	
**Post Menopausal**	N	849	712	P = 0.34^~^
	Yes	547 (64.4%)	476 (66.9%)	
	No	302 (35.6%)	236 (33.1%)	
**Age at Menarche**	N	840	709	P = 0.05^#^
	Median (IQR)	13 (12–14)	13 (12–14)	
**Age at Menopause**	N	485	420	P = 0.38^#^
	Median (IQR)	50 (46–52)	50 (47–52)	
**Number of children**	N	849	712	P < 0.01^#^
	Median (IQR)	2 (1–3)	2 (2–3)	
**Age at first pregnancy**	N	705	646	P < 0.01^#^
	Median (IQR)	24 (21–27)	23 (20–26)	
**Family history of breast cancer**	N	850	712	P < 0.01^~^
	Yes	234 (27.5%)	153 (21.5%)	
	No	616 (72.5%)	559 (78.5%)	

IQR – Inter quartile range;

# – Mann Whitney U test; ~- Chi square test with continuity correction

**Figure 3:**
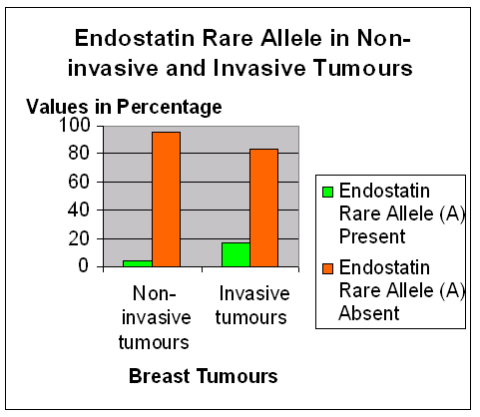
Distribution of the Endostatin Rare Allele (4349A) in patients with non-invasive and invasive tumours. The Rare allele was present in 2 of 44 patients with non-invasive tumours (4.5%) and 130 of 784 patients with invasive tumours (16.6%). Chi square test for trend; p = 0.033.

The observed genotype frequencies of the Endostatin polymorphism are in Hardy Weinberg equilibrium i.e., similar to expected genotype frequencies. The Chi-square goodness of fit statistic (p values at 1 df) was χ^2 ^= 2.71 (p = 0.10). Rare allele carriage rates (combining GA and AA genotypes) were used for further analyses as the numbers with AA genotype were minimal across the different groups. Table 2 shows that the allele carriage rates for this polymorphism is not different between the control and cancer groups and in subgroups classified according to family history and age at diagnosis. In the breast cancer cohort, there were 44 patients with preinvasive tumours. The allele carriage rates is different in the pre-invasive and the invasive cohorts (p = 0.033) (Figure 3). Patients with the A allele have a higher risk of developing invasive breast cancers when compared to the non-invasive group (Odds Ratio = 4.17; 95% CI = 1–17.5). The rare allele carriage rates within subgroups of invasive breast cancer (defined by tumour size, tumour grade, nodal status, vascular invasion and oestrogen receptor status) demonstrated no significant association between the Endostatin polymorphism and breast cancer severity (as measured by the listed prognostic factors) (Table 3). The Endostatin polymorphism did not influence overall survival in patients with invasive breast cancer (N = 568; log rank statistic = 0.52; df = 1; p = 0.47).

**Table 2:** Rare allele carriage rates in breast cancer and controls

**Groups**	**Rare allele (A) carriage rate (%)**		**Odds Ratio (95% CI)**	**Test statistic (p value)^#^**
			
	**Cancer**	**Controls**		
**Overall**	136/846 (16.1%)	115/707 (16.3%)	0.99 (0.75–1.29)	×^2 ^= 0.001; p = 0.97
Positive family history*	38/231 (16.5%)	22/153 (14.4%)	0.17 (0.66–2.07)	×^2 ^= 0.16; p = 0.69
Negative family history*	98/615 (15.9%)	93/554 (16.8%)	0.94 (0.69–1.28)	×^2 ^= 0.1; p = 0.75
Young patients** vs. controls	20/130 (15.4%)	115/707 (16.3%)	0.94 (0.56–1.57)	×^2 ^= 0.01; p = 0.90

# Pearson's chi-square test (with Yates correction where applicable)

*Family history: either first or second-degree relative with breast cancer

**Young cancer patients: </= 50 years of age

**Table 3:** Rare allele carriage rates in subgroups of invasive breast cancer

**Tumour Severity**		**Rare allele carriage rates (%)**	**Test statistic (p value)**^#^
**Tumour size**	Less than 2 cm	68/414 (16.4%)	p = 0.94^$^
	2 to 5 cm	42/270 (15.6%)	
	More than 5 cm	2/17 (11.8%)	
**Tumour Grade**	Grade 1	28/149 (18.8%)	×^2 ^= 1.99; p = 0.37
	Grade 2	57/329 (17.3%)	
	Grade 3	33/239 (13.8%)	
**Nodal Invasion**	Absent	82/498 (16.5%)	×^2 ^= 0.23; p = 0.63
	Present	40/219 (18.3%)	
**Vascular invasion**	Absent	86/515 (16.7%)	×^2 ^= 1.06; p = 0.30
	Present	17/135 (12.6%)	
**Oestrogen Receptor Status**	Present	48/264 (18.2%)	×^2 ^= 0.91; p = 0.34
	Absent	13/98 (13.3%)	

# Pearson's chi-square test (with Yates correction where applicable)

^$ ^Fisher's exact test.

### Endostatin polymorphism and serum Endostatin protein levels

Of the cohort of non smoking healthy peri or post menopausal women (with no history of cancers) attending breast screening, serum Endostatin levels by ELISA and the Endostatin genotype results were available for 57 individuals. The median (inter-quartile range) serum Endostatin levels was 89.5 (82.5–100.5) ng/ml. The genotype distributions in this cohort were also in Hardy Weinberg equilibrium (χ^2 ^= 0.16; p = 0.69). Comparison of the Endostatin levels between the carriers and non carriers of the mutant allele showed no significant difference between groups [MannWhitney U test; z = -0.12; p = 0.90].

### Endostatin polymorphism and protein staining on breast cancer tissue micro array

**Figure 4:**
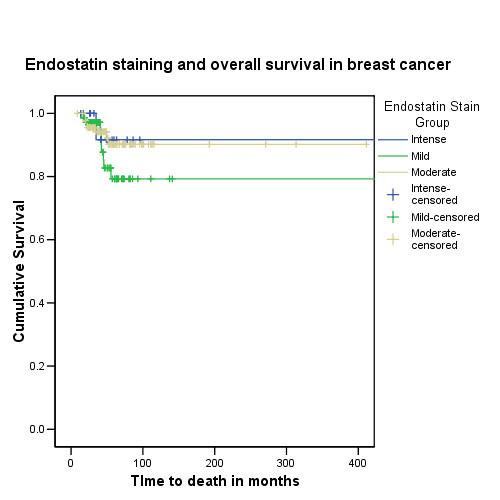
Intensity of Endostatin staining on the breast cancer tissue micro array and overall survival in invasive breast cancer. There were 9 events in 71 patients with mild staining, 7 events in 91 patients with moderate staining and 1 event in 17 patients with intense staining. (Total number = 179; log rank test statistic-1.83; df = 2; p = 0.40)

Following the Endostatin immunostaining of the microarrays, 255 different tumour samples were available for analysis. Figure 2 shows the staining patterns of Endostatin in breast cancer. 8.2% of the tumours showed no staining with Endostatin. The median (inter-quartile range) score on the entire cohort was 4 (3–6). The Endostatin immunostaining scores on the breast cancer tissue micro array showed no association with tumour size (Jonckheere Terpstra observed test statistic = 8265.5; p = 0.35), nodal status (Mann-Whitney U test; z = -0.33; p = 0.74), tumour grade (Jonckheere Terpstra observed test statistic = 10557.5; p = 0.23), vascular invasion (Mann-Whitney U test; z = -0.5; p = 0.62) and oestrogen receptor status (Mann-Whitney U test; z = -0.43; p = 0.66). To assess the impact of Endostatin staining on overall survival, the Endostatin scores were recoded into an ordinal variable comprising three levels: Minimal staining (scores up to 3), moderate staining (from 3.01 to 6.99) and intense staining (from 7 to 9). Survival data and Endostatin scores were available on 179 patients with invasive breast cancer (Figure 4). Although there is no significant association between Endostatin staining and overall survival, the data suggests that tumours with reduced Endostatin staining tend to do worse. The average levels of Endostatin staining were compared between carriers and non carriers of the rare Endostatin allele and no significant difference was found (Mann-Whitney U test; z = -0.52; p = 0.60).

## Discussion

Endostatin is thought to be important in the preventing tumour progression mainly by inhibition of angiogenesis [22]. This has resulted in its evaluation in clinical trials in advanced cancer [23]. The role of the non-synonymous coding Endostatin polymorphism (4349G > A) in exon 42 of the Collagen 18A gene in carcinogenesis has been evaluated in several cancers including that of the prostate [17,24,25], large bowel [26], multiple myeloma [18] and leukaemia [27]. Although the rare allele was initially suggested to be associated with increased prostate cancer risk [17], a recent large study has negated this association [25].

The frequency of the rare allele in our Caucasian control cohort (8.2%) is similar to other Caucasian populations, but much higher than in Black and Chinese populations [17,18,25,27]. This study has not demonstrated a significant association between the polymorphism and breast cancer susceptibility in the UK population. However, within the breast tumour cohort, the rare allele (A) appears to be associated with invasive breast cancers (p = 0.03). The rare allele carriage rates in the non-invasive tumour group (4.5%; n = 44) is significantly lower than the rates in the invasive breast cancer group (16.6%; n = 784) and in the control group (n = 16.3%; n = 707). It is well recognised that angiogenesis and vascular stroma formation precedes the development of invasive breast disease [28,29]. Only a proportion of women with DCIS will however progress to invasive disease [30] and the mechanisms underlying this transformation are still unclear. The finding therefore of a genetic variant predisposing to invasion needs further consideration as the role of Endostatin as an inhibitor of tumour angiogenesis and thereby growth is well recognised. Among those with invasive breast cancer, there is no association between the polymorphism and standard prognostic factors such as tumour size, tumour grade, axillary lymph nodal status, vascular invasion and oestrogen receptor status. However, we also acknowledge that this association would not be significant if subjected to correction for multiple testing and needs further investigation in a larger cohort of patients and in different populations.

A study reported recently [31] on the Brazilian population has shown that the AA polymorphism may be associated with increased breast cancer risk, although the risk in heterozygotes were lower than the risk in GG homozygotes. No other association was found between the Endostatin polymorphism and pathological features including histological type, grade, ER and PR status and TNM stage. The study also did not find any relationship between the Endostatin genotype and serum levels [31]. The limitations of this study however were the inclusion of both genders and different races, which has the potential for population stratification.

We have not shown any effect of the Endostatin polymorphism on serum levels in healthy older women, which is in accordance with previous reports and with the hypothesis that the mutant protein has an altered function but a stable structure [17]. Endostatin protein expression on an invasive breast cancer tissue micro array has also shown no association with the Endostatin polymorphism or with standard prognostic markers in breast cancers (tumour size, tumour grade, nodal status, vascular invasion and oestrogen receptor status). We also found no association with overall survival, but in view of the reduced length of follow up information and the rarity of the end points (death), larger numbers of patients are required to demonstrate any significant effect on survival. The fact that the Endostatin staining did not correlate with other prognostic markers may suggest that Endostatin could have an independent effect on survival. We acknowledge that estimating 'protein levels' in tumour tissue by Western blot analyses may have provided information of more value that that obtained semi-quantitatively by Immunohistochemistry.

## Conclusion

This study has shown no significant association between the 4349G > A coding non-synonymous Endostatin polymorphism and breast cancer susceptibility. The polymorphism is also not associated with tumour severity in patients with invasive breast cancer. We have however shown for the first time that Endostatin genotype may be associated with progression to invasive breast disease. Although the polymorphism is not associated with Endostatin levels in serum and tumour tissue, it may potentially affect Endostatin function. Further studies exploring the differences in genotype frequencies among those with non-invasive and invasive cancer and expression patterns in DCIS are needed.

## Competing interests

The author(s) declare that they have no competing interests.

## Authors' contributions

SPB drafted the manuscript, carried out patient recruitment and with JG carried out the experiments. SSC reviewed the pathology, constructed the micro array and assessed the micro array staining. SPB and AC participated in the design of the study and performed the statistical analysis. MWR and NJB conceived the study, and participated in its design and coordination and helped to draft the manuscript. All authors read and approved the final manuscript.

## Pre-publication history

The pre-publication history for this paper can be accessed here:


